# Supplementary figures and images for: SPHK1/S1PR1/PPAR-α axis restores TJs between uroepithelium providing new ideas for IC/BPS treatment
Source: Life Sci Alliance. 2024 Nov 22;8(2):e202402957. doi: 10.26508/lsa.202402957 (PMC11584326; doi:10.26508/lsa.202402957)

Figure S2B

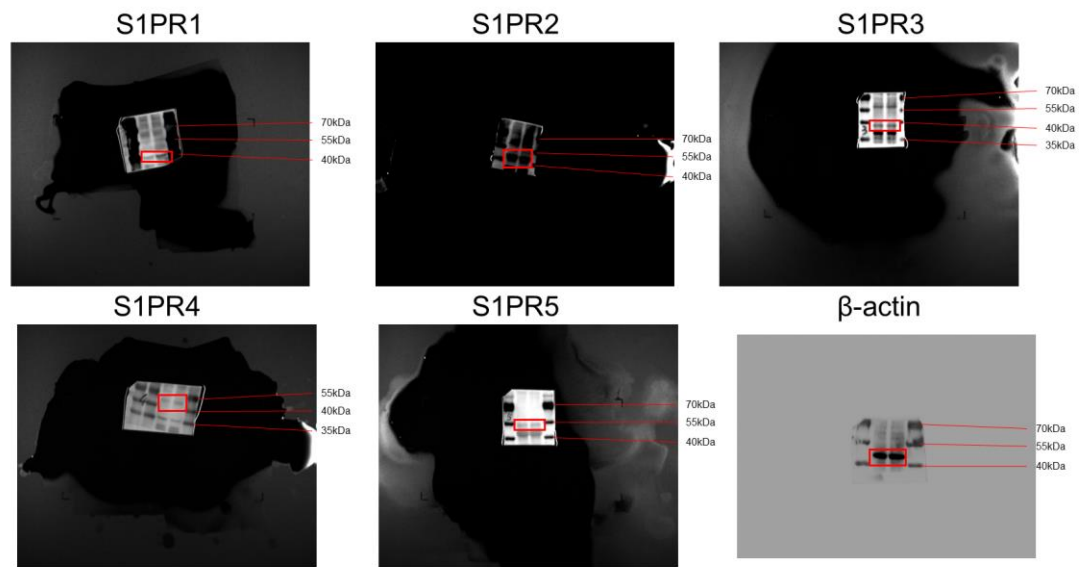

Figure S2C

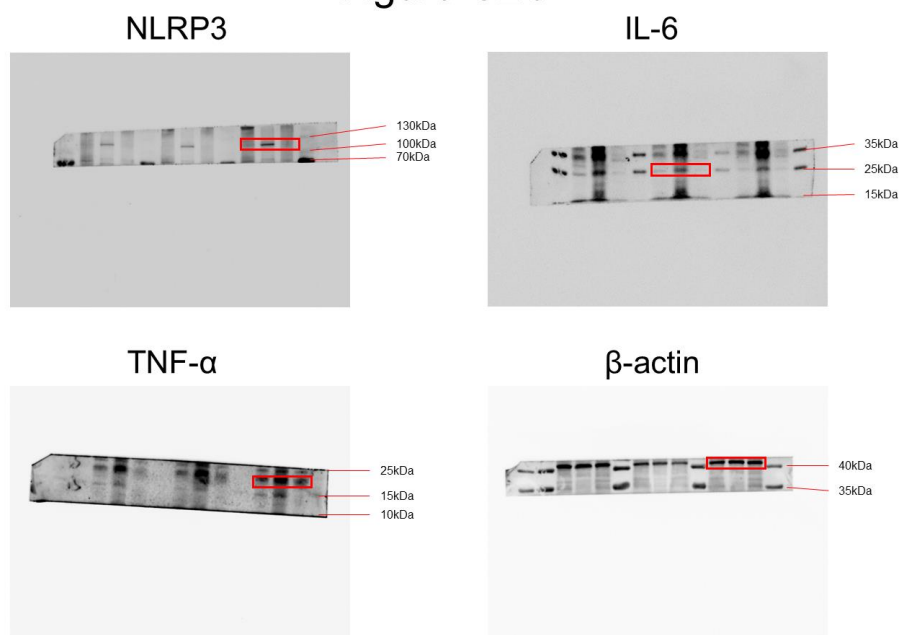

Supplement: Supplementary file 1 [file LSA-2024-02957_SdataFS1.pdf]

Figure 2A

ZO-1

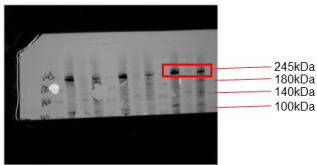

E-cad

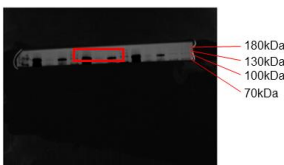

Occludin

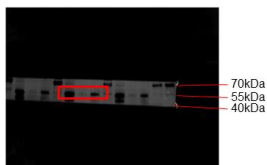

Claudin-4

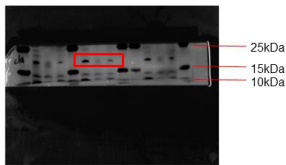

$\beta$ -actin

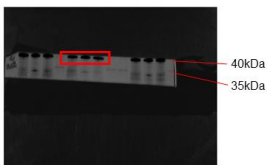

Supplement: Supplementary file 2 [file LSA-2024-02957_SdataF2.pdf]

Figure 3G

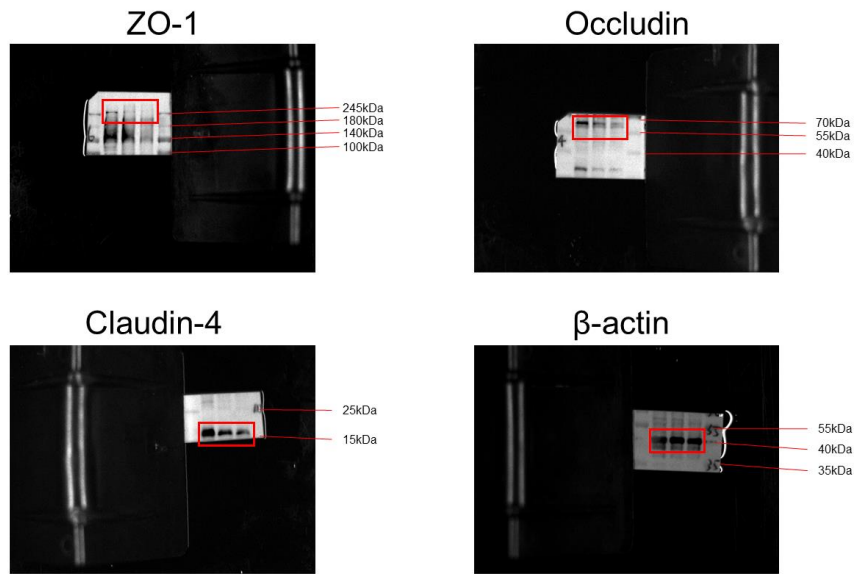

Supplement: Supplementary file 3 [file LSA-2024-02957_SdataF3.pdf]

Figure 5A

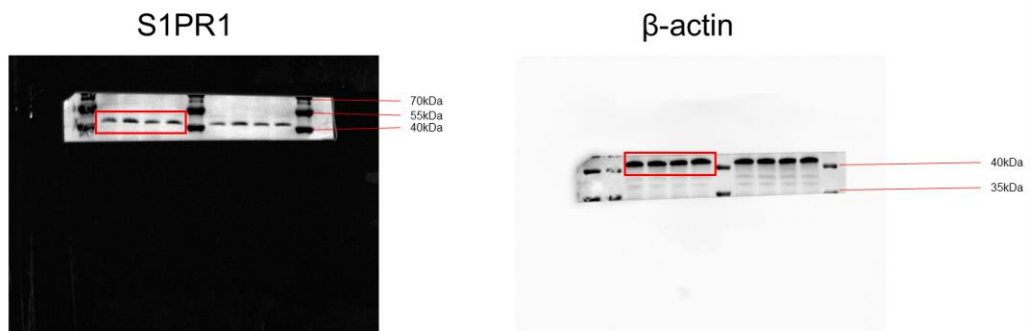

Figure 5B

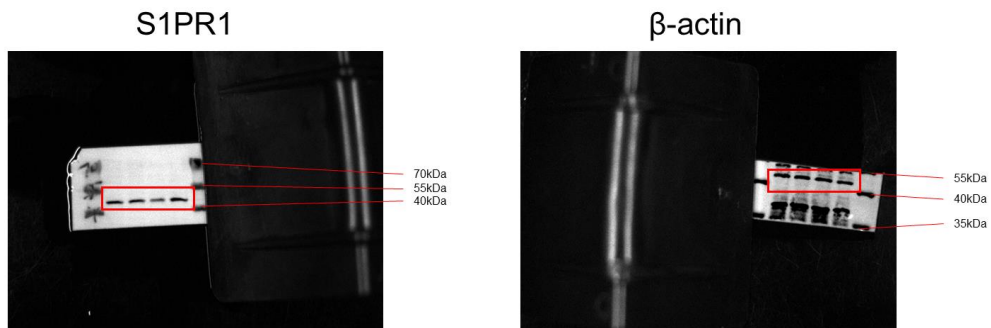

Figure 5D

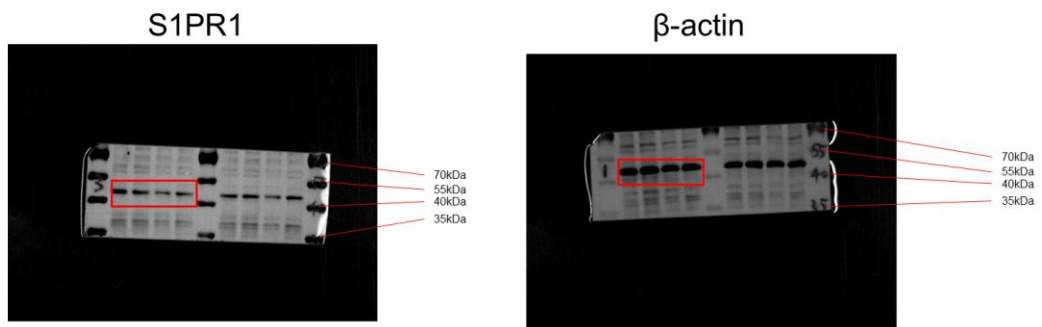

Figure 5E

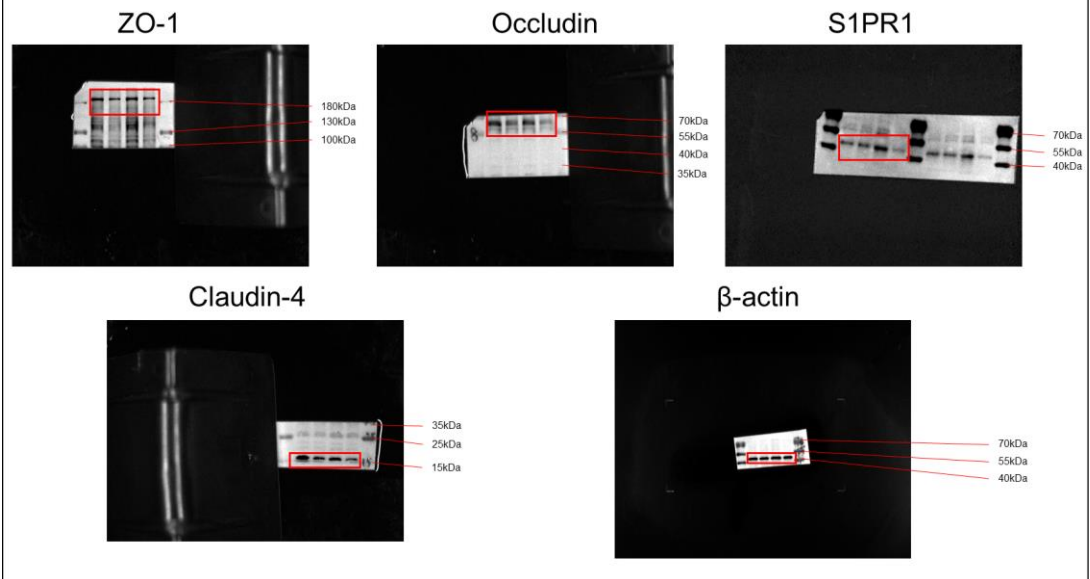

Supplement: Supplementary file 5 [file LSA-2024-02957_SdataF5.pdf]
