# Supplementary material for: SPHK1/S1PR1/PPAR-α axis restores TJs between uroepithelium providing new ideas for IC/BPS treatment
Source: Life Sci Alliance. 2024 Nov 22;8(2):e202402957. doi: 10.26508/lsa.202402957 (PMC11584326; doi:10.26508/lsa.202402957)
Supplement: Supplementary file 4 [file LSA-2024-02957_SdataF4.pdf]

Figure 4A

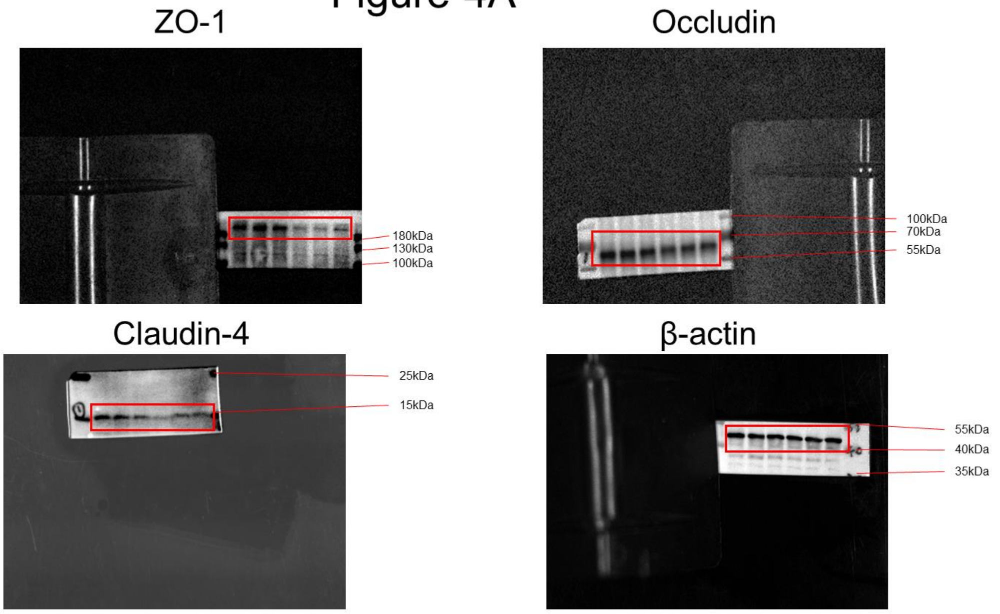

Figure 4C

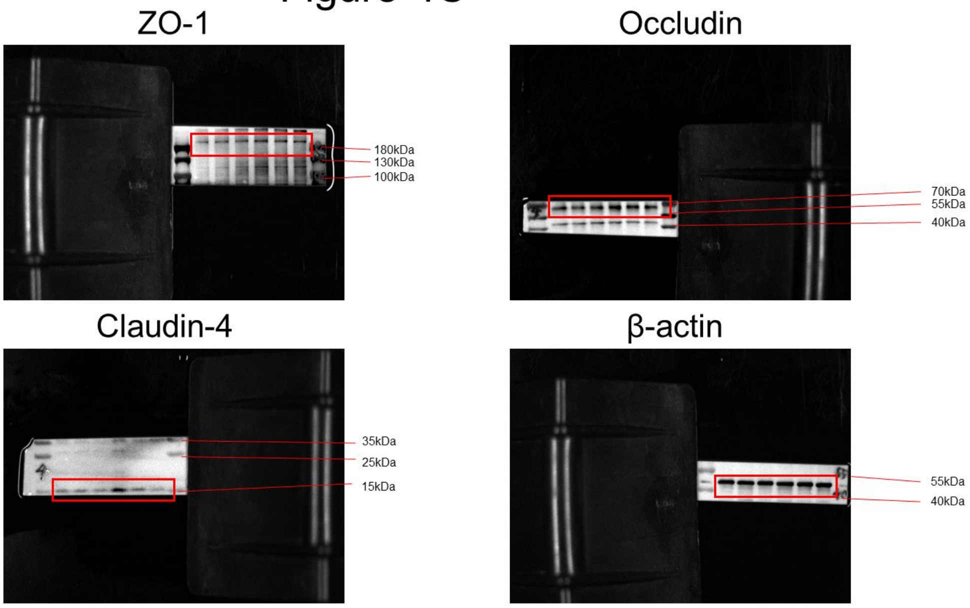

Figure 4E

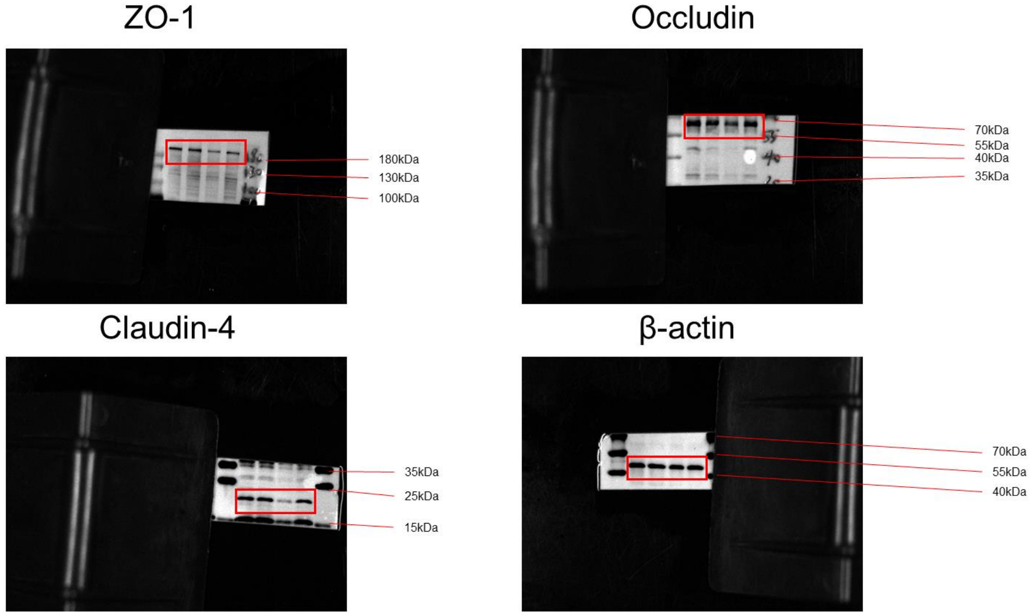

Figure 4I

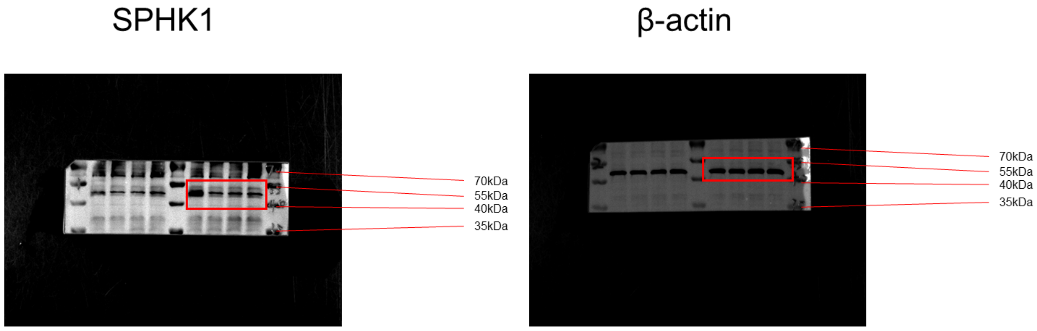

Figure 4J

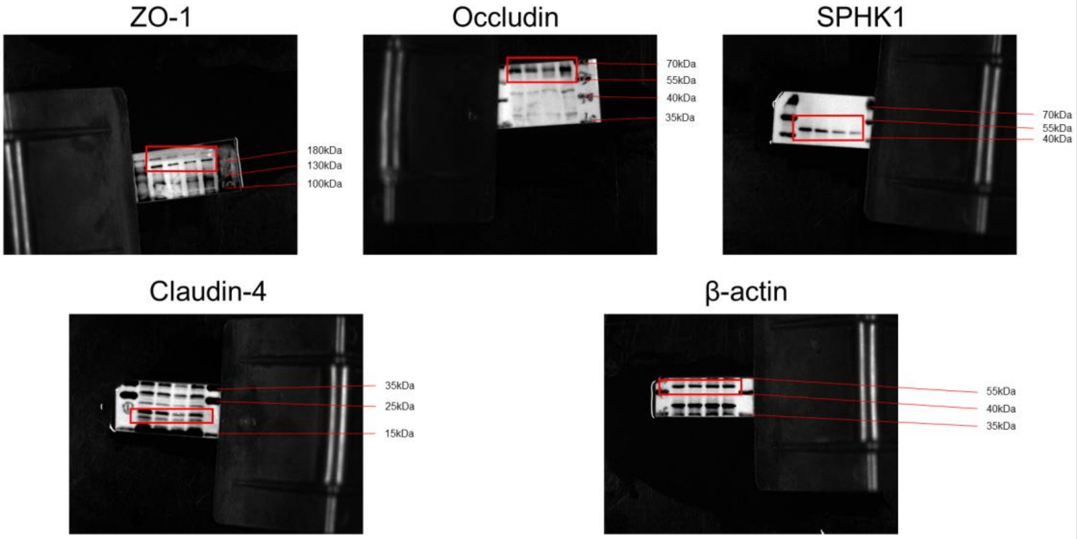

ZO-1
180kDa
130kDa
100kDa
Occludin
70kDa
55kDa
40kDa
35kDa
SPHK1
70kDa
55kDa
40kDa
Claudin-4
35kDa
25kDa
15kDa
β-actin
55kDa
40kDa
35kDa
