# Supplementary material for: SPHK1/S1PR1/PPAR-α axis restores TJs between uroepithelium providing new ideas for IC/BPS treatment
Source: Life Sci Alliance. 2024 Nov 22;8(2):e202402957. doi: 10.26508/lsa.202402957 (PMC11584326; doi:10.26508/lsa.202402957)
Supplement: Supplementary file 6 [file LSA-2024-02957_SdataF6.pdf]

Figure 6A

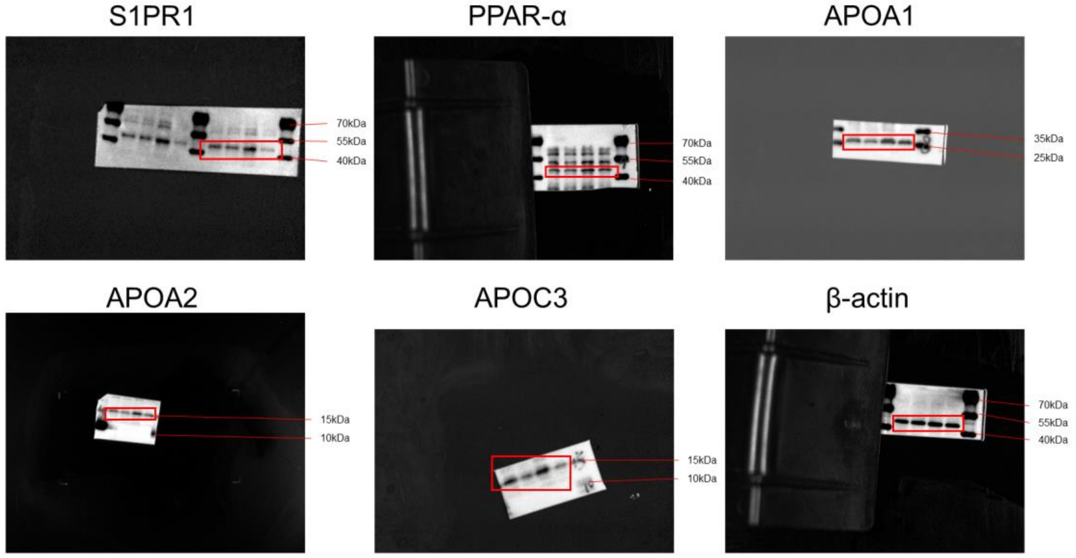

Figure 6B

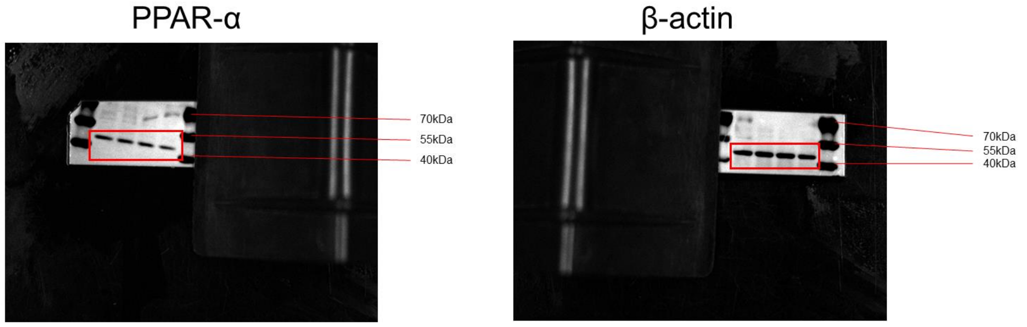

Figure 6C

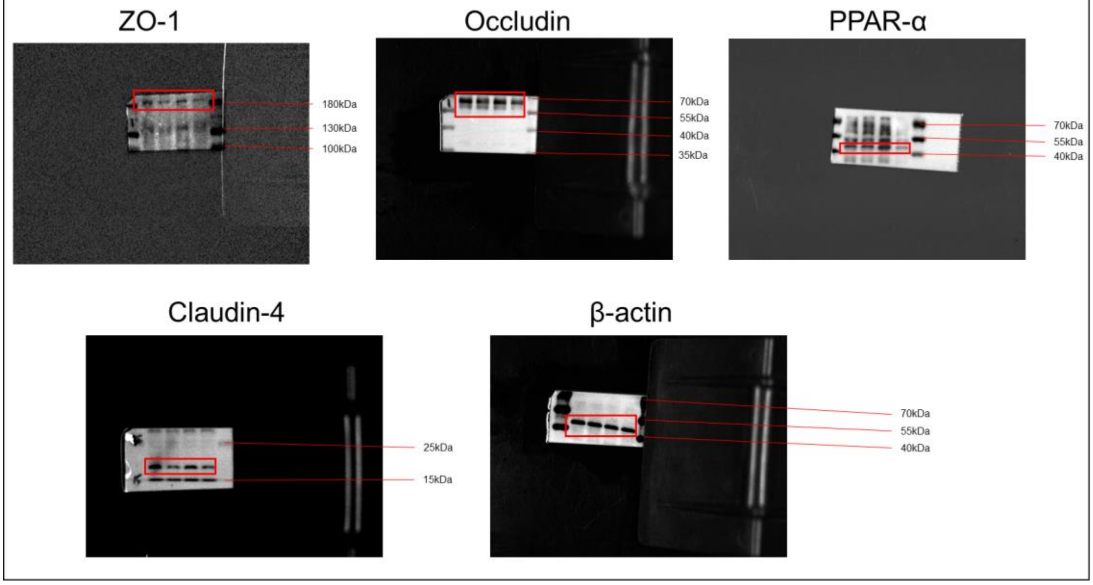

Figure 6F

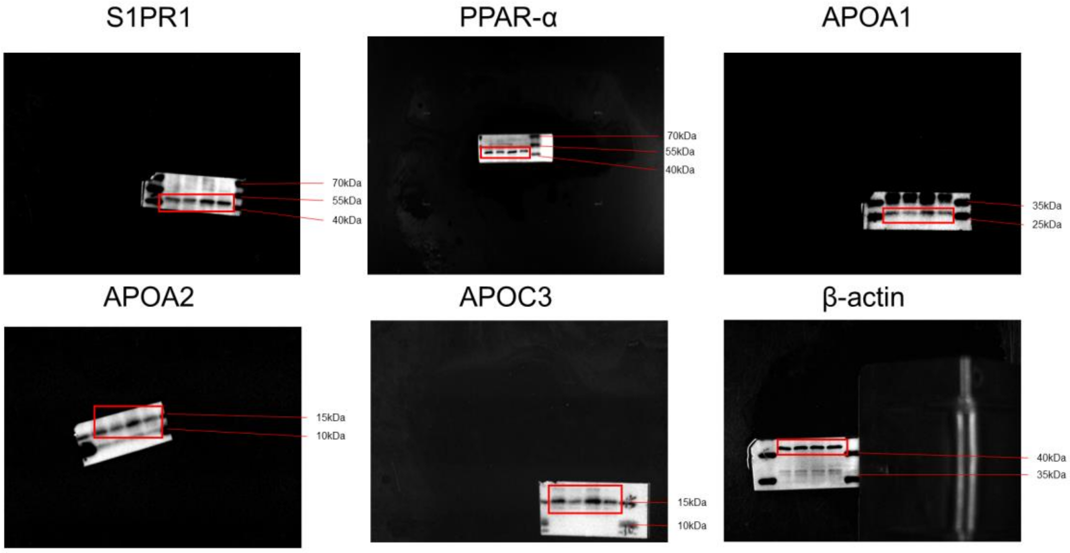

Figure 6G

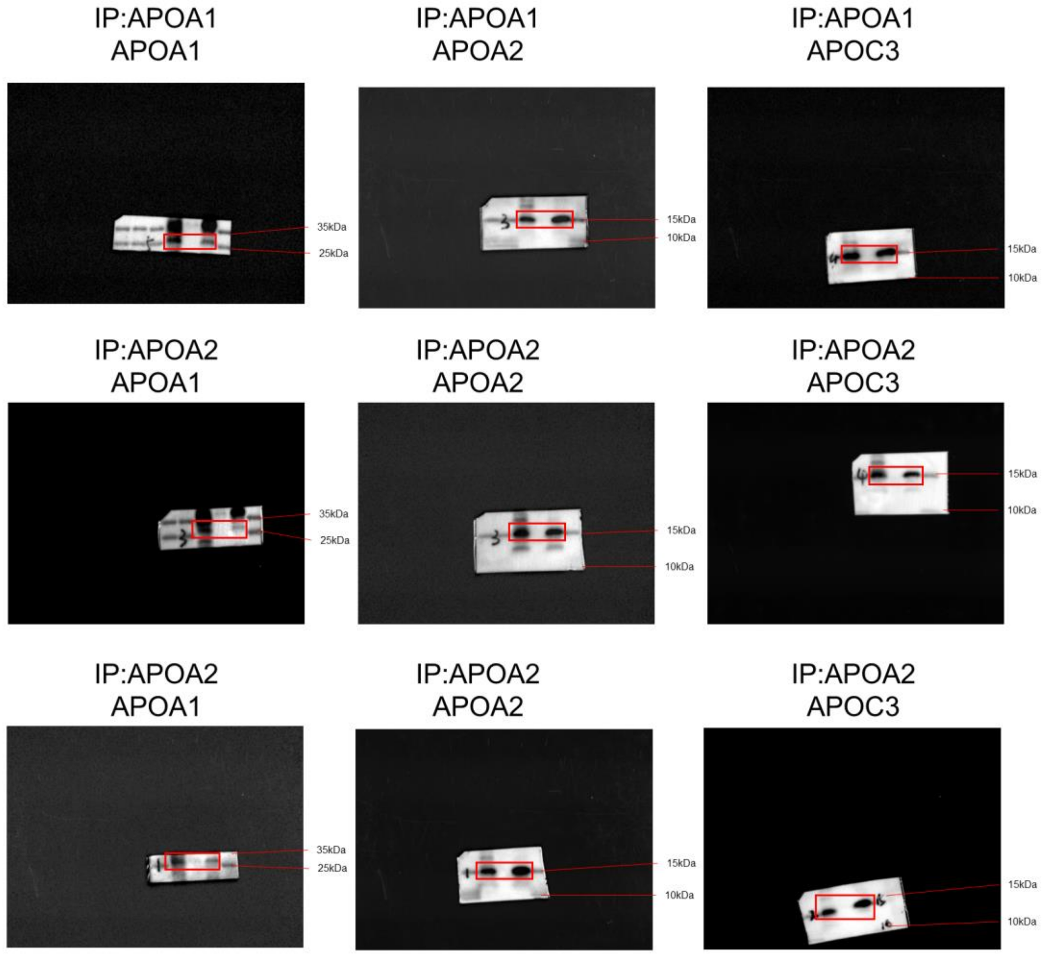
IP:APOA1
APOA1
35kDa
25kDa
IP:APOA1
APOA2
15kDa
10kDa
IP:APOA1
APOC3
15kDa
10kDa
IP:APOA2
APOA1
35kDa
25kDa
IP:APOA2
APOA2
15kDa
10kDa
IP:APOA2
APOC3
15kDa
10kDa
IP:APOA2
APOA1
35kDa
25kDa
IP:APOA2
APOA2
15kDa
10kDa
IP:APOA2
APOC3
15kDa
10kDa
